# Short-Term Effects of Optically Induced and Digitally Simulated Myopic Blur on Retinal Perfusion: Insights From Optical Coherence Tomography Angiography

**DOI:** 10.1167/iovs.67.6.13

**Published:** 2026-06-08

**Authors:** Hosein Hoseini-Yazdi, Scott A. Read, Fan Yi, David Alonso-Caneiro, Michael J. Collins

**Affiliations:** 1 Contact Lens and Visual Optics Laboratory, Centre for Vision and Eye Research, Optometry and Vision Science, Queensland University of Technology, Brisbane, Queensland, Australia; 2 School of Science, Technology and Engineering, University of the Sunshine Coast, Petrie, Queensland, Australia

**Keywords:** blur, myopia, myopic defocus, optical coherence tomography angiography, simulated blur

## Abstract

**Purpose:**

To examine changes in retinal perfusion density (PD) following short-term exposure to optically induced myopic defocus, an ocular growth-inhibitory stimulus, and achromatic digitally simulated myopic blur, an ocular growth stimulus.

**Methods:**

The left eyes of 12 emmetropic and 12 myopic healthy young adults (mean ± SEM age: 24 ± 1 years) underwent optical coherence tomography angiography to assess retinal PD before and following 60 minutes of blur exposure. Optical myopic defocus was induced by viewing a video through +2 D lenses, digitally simulated myopic blur by viewing the same video filmed with a +2 D defocused camera, and the control (no-blur) condition by viewing the original video through optimal refractive correction. PD changes were examined using linear mixed models, with the control-condition changes as a covariate.

**Results:**

A significant blur by eccentricity interaction was observed (*P* < 0.001), with the overall retinal PD increasing with optical myopic defocus compared to simulated myopic blur in the fovea by 2.9% ± 1.0% (*P* = 0.003) but decreasing in the outermost 2- to 3-mm parafovea by −2.3% ± 0.5% (*P* < 0.001). Significant PD changes were also observed associated with the blur condition, perfusion layer, eccentricity, and refractive status (interaction, *P* = 0.04). The most pronounced effects were observed in the foveal deep capillary plexus, where emmetropes exhibited increased PD during optical myopic defocus and decreased PD during simulated myopic blur (3.8% ± 2.3% vs. −6.4 ± 2.2%; *P* < 0.001), but myopes exhibited significantly different responses compared with emmetropes to both the optical blur (−4.0% ± 2.3%; *P* = 0.035) and the simulated myopic blur (0.7% ± 2.3%; *P* = 0.046).

**Conclusions:**

Significant blur-driven changes were observed in the retinal PD of emmetropic eyes, increasing with optical myopic defocus but decreasing with simulated myopic blur of comparable magnitude, particularly in the foveal deep capillary plexus, with these responses not observed in myopic eyes. These bidirectional retinal perfusion responses are unlikely to be driven solely by blur magnitude or contrast modulations and are more plausibly related to vergence cues that were present with optical myopic defocus but absent in digitally simulated myopic blur.

Early studies in animals demonstrated that the eye not only perceives retinal image blur but also responds to blurred images by increasing its axial growth and developing myopia.^1^^,^^2^ This blur-induced myopia, commonly referred to as form deprivation myopia, is thought to result from selective contrast reductions at high spatial frequencies^3^^,^^4^ and may be inhibited by enhancing contrast at high spatial frequencies.^5^ Myopia is also induced by hyperopically defocused images, in which the image is formed behind the retina by negative lenses, while contrast is selectively reduced at high spatial frequencies.^6^^,^^7^ However, myopically defocused images, in which the image is formed in front of the retina by positive lenses or during recovery from form deprivation myopia, result in slower eye growth and hyperopia,^7^^,^^8^ despite also producing selective contrast reductions at high spatial frequencies.^9^

Human eyes have exhibited similar short-term blur-induced responses, including increased axial length with brief exposures to diffusely blurred (by Bangerter foils),^10^ digitally blurred (by low-pass filtering of achromatic images),^11^^,^^12^ or hyperopically defocused images,^10^^,^^13^ and decreased axial length with brief exposures to myopically defocused images.^10^^–^^14^ Long-term clinical studies have also demonstrated that ocular growth is altered with optical interventions that induce retinal image blur^15^ or alter peripheral retinal image contrast.^16^ Specifically, optical methods that impose myopic defocus on the retina slow ocular growth in myopic children,^17^^–^^20^ whereas those imposing hyperopic defocus on the retina promote ocular growth in hyperopic children.^21^ These studies have collectively provided strong evidence supporting vision-driven mechanisms regulating growth and refractive development of the eye, with retinal image quality and sign of defocus playing critical roles.^22^

The exact mechanisms underlying blur-mediated eye growth are not fully understood. However, they are thought to largely occur locally within the eye, involving a cascade of signals stemming from the retina that influence the choroid and ultimately induce scleral remodeling, thereby determining eye size.^22^^,^^23^ Supporting evidence is provided by animal studies showing that form deprivation myopia is induced when only part of the retina is exposed to degraded images,^24^ accompanied by a localized disruption in retinal electrophysiological responses^25^ and reduced retinal dopamine.^26^ Similarly, localized changes in choroidal thickness have been reported in the human eye in response to localized optical defocus stimuli, suggesting the presence of local mechanisms of eye growth.^27^^,^^28^ Longitudinal studies have also demonstrated that reduced inner retinal neuronal responses are associated with development^29^ and greater progression^30^ of myopia in children. Overall, these studies suggest that retinal neuronal responses are modulated locally by both image quality and the sign of defocus and regulate eye growth and the development of refractive errors.

It is expected that blur-induced modulation of retinal neuronal responses produces corresponding changes in retinal blood flow through neurovascular coupling mechanisms to meet metabolic demands.^31^ However, there is limited understanding regarding these blur-induced retinal vascular changes. Using functional magnetic resonance imaging, Duong et al.^32^ reported localized increases in ocular blood flow in the cat eye in response to hemifield retinal stimulation with high-contrast gratings, but they were unable to differentiate retinal from choroidal blood flow changes. More recently, the effect of short-term imposed myopic defocus on chorioretinal blood flow was examined in human eyes using laser speckle flowgraphy (LSFG).^33^ However, blur-induced blood flow changes in the macula were observed only in the choroid and not in the retina, likely because any blur-driven changes in retinal blood flow were masked by the dominant signal from the choroidal blood flow. Nevertheless, retinal flow measurements obtained from the peripapillary region in this study did not show significant changes with myopic defocus.^33^

Optical coherence tomography angiography (OCTA) has emerged as a non-invasive imaging modality providing high-resolution depth-resolved measurements of retinal perfusion.^34^ Retinal perfusion can be assessed across the superficial vascular plexus (SVP), as well as the intermediate capillary plexus (ICP) and deep capillary plexus (DCP), each supplying different retinal neuronal populations. Consequently, OCTA has the potential to assess blur-induced changes across different perfused retinal layers, thereby providing insights into the underlying vascular and neuronal mechanisms.

In this study, OCTA was employed to examine retinal perfusion changes in healthy young adults during short-term exposure to image blur, either optically induced by positive lenses to create myopic defocus on the retina or digitally generated to produce comparable achromatic simulated myopic blur. Optical and simulated myopic blur were included in this study to determine whether optical vergence cues, present only in optical myopic defocus, provide distinct signals to the eye beyond the blur signal, which is present in both blur conditions, and thereby differentially influence retinal perfusion. It should also be noted that achromatic simulated blur was used in the present study, without manipulation of chromatic aberrations, which differs from other approaches employing chromatically simulated blur, designed to mimic the chromatic aberration profile associated with myopic defocus and shown to demonstrate anti-myopiagenic effects.^35^^,^^36^

Blur-induced changes in retinal perfusion were examined across the SVP, ICP, and DCP layers at different macular eccentricities and compared between emmetropic and myopic eyes to provide insights into potential refractive error-dependent regional vascular mechanisms underlying these blur-driven responses.

## Methods

### Participants

Twenty-four healthy young adults between the ages of 18 and 35 years participated in this study. They all exhibited good general health, normal best-corrected visual acuity of 0.00 logMAR or better in each eye, normal binocular vision, stable fixation, and no history or evidence of amblyopia, strabismus, accommodation dysfunction, or any other significant ocular disease, injury, or surgery. Participants who smoked or who were using pharmacological or optical treatments to control the progression of myopia (including atropine, multifocal spectacle and contact lenses, and orthokeratology) were not included. Normal anterior and posterior segments were confirmed through slit-lamp examination and optical coherence tomography, respectively.

Eligible participants had spherical equivalent refraction (SER) between +0.75 D and −6.00 D, astigmatic refractive errors of less than 1.75 D in each eye, and less than 1.00 D of anisometropia, determined using a non-cycloplegic subjective refraction. Based on refraction of the left eye, 12 participants were classified as emmetropes with SER between −0.25 D and +0.75 D, and 12 participants were classified as myopes with SER between −0.50 D and −6.00 D. Habitual rigid contact lens wearers were excluded, whereas habitual soft contact lens wearers were asked to use their single-vision spectacle correction instead of soft contact lenses on the day of testing. Eligible participants underwent ocular biometry using the Lenstar LS 900 (Haag Streit AG, Köniz, Switzerland) in the screening visit. The resulting measurements of central corneal thickness, corneal curvature, anterior chamber depth, crystalline lens thickness, and axial length were used along with the refractive error for adjusting the lateral magnification of OCTA images, using our previously described methods,^37^^,^^38^ utilizing Bennett's method to determine the equivalent power of the eye and crystalline lens.^39^ Therefore, comparable retinal regions relative to the fovea were examined across both refractive groups and blur conditions. Ethics approval was obtained from the Queensland University of Technology Human Research Ethics Committee (approval no. 8809). Written informed consent was obtained from the participants, and the study was conducted in accordance with the tenets of the Declaration of Helsinki.

### Short-Term Induced Blur Conditions

**Figure 1.**
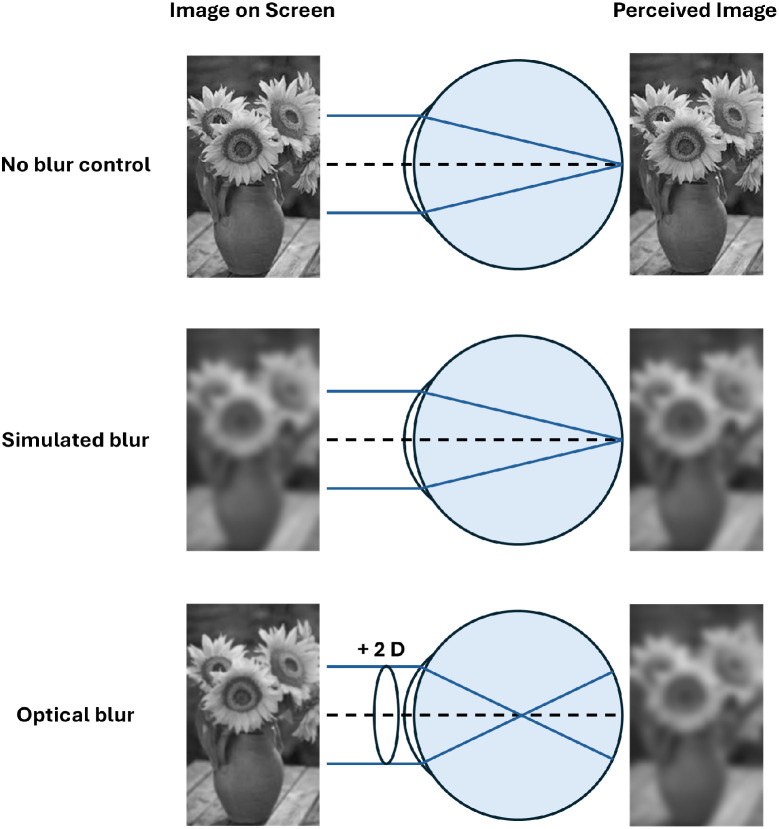
Overview of the blur conditions to which both eyes were exposed for 60 minutes in separate, randomly ordered study visits. In the no-blur control condition (*top row*), a movie with original high-definition quality was displayed on the screen and perceived as a clear image through optimal spherocylindrical correction of both eyes. In the digitally simulated myopic blur condition (*middle row*), the movie displayed on the screen was blurred, as it was filmed with a digital camera fitted with a 6-mm artificial pupil and a +2 D spherical trial lens, and it was perceived as a blurred image despite optimal spherocylindrical correction of both eyes. In the optical blur condition (*bottom row*), the movie displayed on the screen was slightly blurred, as it was filmed with the same digital camera fitted with a 6-mm artificial pupil and a plano spherical trial lens, and it was perceived as a blurred image because a +2 D spherical lens was added to the optimal spherocylindrical correction of both eyes.

Eligible participants attended three separate study visits, during which both their eyes were exposed for 60 minutes to one of three blur conditions, including a no-blur control, +2 D equivalent simulated blur, or +2 D of optical blur (Fig. 1). For each participant, study visits were conducted on separate days, in randomized order. In the no-blur control condition, participants watched a movie in its original high-definition quality on a high-resolution liquid-crystal display (LCD) monitor at a distance of 4 meters (28-inch UE590 UHD 4K monitor, 3840 × 2160 resolution, 60-Hz refresh rate, subtending visual angles of 9° horizontally and 5° vertically; Samsung, Suwon, South Korea), with their optimal spherocylindrical distance correction in a trial frame (i.e., a clear movie). However, during the simulated myopic blur condition, they wore their optimal spherocylindrical distance correction in both eyes but viewed a blurred version of the movie. The blurred movie was created previously by recording the original movie with a digital camera fitted with a 6-mm artificial pupil and a +2 D spherical trial lens while the auto-focus function of the camera was deactivated. In contrast, induced optical blur was achieved by inserting an additional +2 D trial lens in front of the optimal spherocylindrical correction of both eyes while the participants watched the clear movie (i.e., optically blurred). Therefore, both eyes were exposed to the no-blur control condition and optical blur across the entire visual field but were exposed to simulated myopic blur across only the central 9° horizontally and 5° vertically of the visual field. However, it should be noted that the peripheral retina primarily viewed a blank wall surrounding the LCD monitor, which contained limited spatial detail and low contrast. Consequently, any differences in peripheral blur signals arising from spatial details in surrounding scene across conditions are unlikely to have substantially influenced retinal perfusion.

Because the quality of the simulated myopic blur movie was modulated by both optical properties of the digital camera and the added +2 D spherical lens, the camera-based optical modulations were also accounted for during the optical blur condition by displaying a movie recorded with the same digital camera equipped with a 6-mm artificial pupil but fitted with a plano trial lens (i.e., in-focus). Therefore, the visual perception was similar between the simulated and optical myopic blur conditions, with image blur produced either by degrading the quality of the displayed movie (simulated myopic blur) or by altering the incident light angles on the retina (optical myopic blur), respectively. Any potential optical magnification effects are expected to be comparable between the two blur conditions, as both were induced using +2 D lenses, placed either in front of the eye (optical myopic blur) or in front of the digital camera (simulated myopic blur). To assess the visual effects of the blur conditions, monocular visual acuity of the left eye was measured before and then at the beginning of exposure to each blur condition using the Freiburg visual acuity test (FrACT) computerized chart,^40^ which was displayed on the same LCD monitor and modified for each blur condition in the same way as the movie stimuli.

Potential confounding effects of chromatic cues^32^^,^^41^^,^^42^ and caffeine intake^43^ on retinal perfusion were minimized by displaying movies in grayscale and asking participants to avoid caffeinated drinks during at least 2 hours prior to the start of each study visit. Diurnal variations in retinal perfusion^44^ were also controlled by conducting all study visits at approximately the same time of day. The average study visit start times were 12:28 ± 00:27 for the no-blur control, 12:47 ± 00:26 for the simulated myopic blur, and 12:34 ± 00:27 for the optical blur condition. The right untested eye was open during viewing periods (optimally corrected during control and simulated myopic blur visits and blurred by an additional +2 D lens during the optical myopic blur visit) but occluded during ocular measurements of the left tested eye.

### Procedures

Each study visit began with a 10-minute period of viewing a grayscale movie at 4 meters under ∼10 lux ambient illumination while wearing optimal spherocylindrical correction for the viewing distance (with a +0.25 D incorporated) in a trial frame. This period served to dissipate potential effects from prior near work or outdoor activities on retinal perfusion.^45^^–^^47^ Baseline measurements of monocular visual acuity and retinal perfusion of the left eye were then obtained, using the FrACT test and OCTA, respectively. During OCTA imaging, the participants fixated on a high-contrast feature within a grayscale still image displayed on a mobile phone screen (Samsung Galaxy S7), with spherocylindrical refractive errors optimally corrected through a Badal system viewed through a cold mirror mounted on the objective lens of the OCTA device, as described in detail in our previous studies.^27^^,^^45^^,^^48^ Participants then underwent the assigned blur condition for 60 minutes, viewing the corresponding movie with the appropriate trial lenses in place (Fig. 1), with measurements of the monocular visual acuity of the left eye obtained at the beginning of the blur period (over approximately 3 minutes), before any significant blur adaptation had taken place.^49^ Immediately after the 60 minutes of exposure to blur, retinal perfusion was measured again using OCTA while the left eye remained exposed to the assigned blur stimulus in the Badal system and fixated on a high-contrast feature within the corresponding grayscale still image displayed on the mobile phone screen.

The still image presented through the Badal system during OCTA imaging was similar across all measurement time points; however, its visual quality depended on the blur condition being tested. Therefore, a high-definition original version of the still image was used during all baseline measurements and during the 60-minute measurement in the no-blur control visit. A recorded blurred version of the image was used during the 60-minute measurement in the simulated myopic blur visit, whereas a recorded clear version was used during the 60-minute measurement in the optical blur visit (and viewed through +2 D optical blur placed in the Badal system). Furthermore, these images were size matched to the spatial details shown on the LCD monitor at 4 meters during the 60-minute blur period, with adjustments made to the image size to account for the magnification of the Badal system (11× relative to free-space viewing).^50^

### Retinal Perfusion

OCTA imaging was carried out using the SPECTRALIS device (Heidelberg Engineering, Heidelberg, Germany) for non-invasive assessment of the retinal perfusion. A single set of OCTA scans was acquired at baseline and again after 60 minutes of exposure to each blur condition, covering a 15° × 15° (nominally 4.4 × 4.4 mm) area, centered on the fovea. Each OCTA volume consisted of 384 horizontal B-scans, spaced 11 µm apart, with each B-scan comprised of 384 A-scans and averaged five times using the automatic real time tracking (ART-5) feature of the device. The follow-up mode of the instrument was used to ensure that retinal perfusion was examined across identical regions across all measurement times. OCTA images were reviewed for motion artifacts immediately following acquisition and repeated if significant artifacts were identified. During all OCTA image acquisitions, the flickering blue internal fixation target of the SPECTRALIS was switched off to minimize any potential localized changes in the foveal perfusion associated with flickering light.^32^

**Figure 2.**
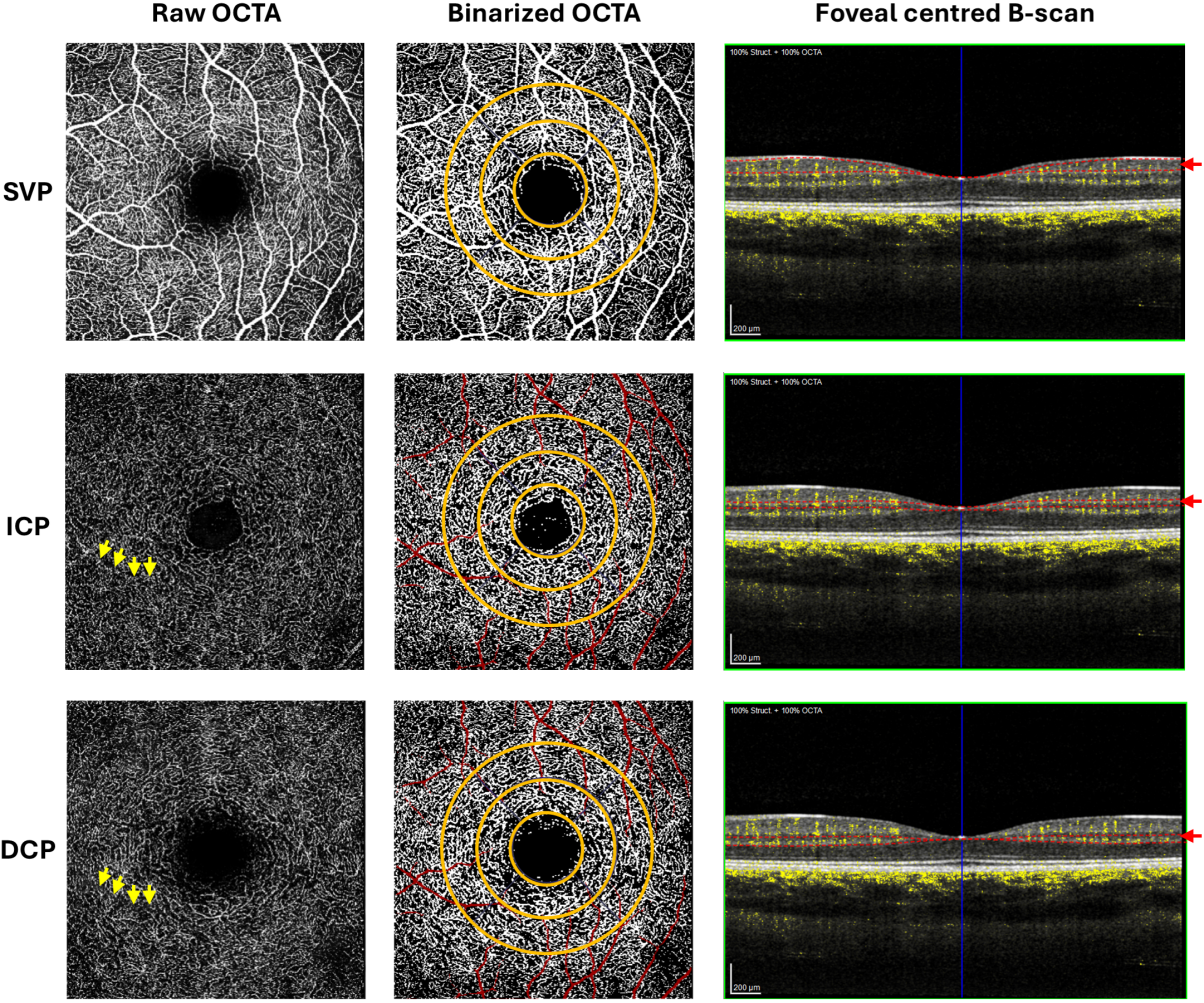
Representative OCTA images, nominally covering 4.4 × 4.4 mm of the central macula, showing raw (*left column*) and binarized (*middle column*) images derived from different retinal slabs positioned at the instrument's pre-defined depths (*red arrows*, *right column*). The OCTA images demonstrate the superficial vascular complex (SVP, *top row*), ICP (*middle row*), and DCP (*bottom row*). Projection artifacts caused by large superficial retinal vessels were visible in the raw OCTA images of the ICP and DCP, despite using the instrument's automatic PAR (*yellow arrows*). These artifacts were corrected in the binarized OCTA images by identifying and removing the large superficial retinal vessels using custom software (*red vessels* in binarized images). Retinal perfusion was quantified within the central 3-mm region, which was divided into three eccentricities as outlined in *orange* on the binarized OCTA images (*middle column*). Eccentricity zones include the central 1-mm fovea, and the surrounding inner (1- to 2-mm annulus) and outer (2- to 3-mm annulus) parafovea.

After image acquisition, each OCTA image was carefully reviewed to verify accurate automatic boundary segmentation of the retinal slabs, but no manual adjustments were necessary. Projection artifacts caused by superficial large retinal vessels were then removed from deeper retinal slabs both using the instrument's built-in projection artifact removal (PAR) and additional custom software developed in MATLAB R2024a (MathWorks, Natick, MA, USA) to eliminate residual artifacts (Fig. 2).

Retinal perfusion densities (PDs) were then quantified at each of the predefined retinal slabs of the instrument, following the guidelines from Campbell et al.^51^ The SVP slab extended across the ganglion cell layer and the inner 80% of the inner plexiform layer. The ICP slab extended across the outer 20% of the inner plexiform layer and the inner half of the inner nuclear layer. The DCP slab extended across the outer half of the inner nuclear layer and the outer plexiform layer (Fig. 2). Custom software was used to binarize the OCTA images using the Sauvola thresholding method,^52^ with the PD of SVP, ICP, and DCP calculated as the ratio of the area occupied by blood vessels (white pixels) to the total area of the binarized image.

The binarized OCTA images were further reviewed by an observer masked to the blur condition and the participant's refractive status. The PD data from regions affected by poor image quality, particularly due to shadow or line artifacts, were excluded from analysis. This initial visual data cleaning resulted in the exclusion of 8.7% of PD measurements across the SVP, ICP, and DCP of all participants, regions, blur conditions, and measurement time points. Following transverse magnification adjustment of OCTA images, the mean transverse OCTA scan length was 4.20 ± 0.12 mm (range, 4.00–4.40) in emmetropes, and 4.48 ± 0.27 mm (range, 4.10–4.90) in myopes. The retinal PD for each slab was quantified across different eccentricities, including the central 1-mm fovea (∼3°) and the surrounding inner (1- to 2-mm annulus, ∼3°–7°), and outer parafovea (2- to 3-mm annulus, ∼7°–10°) (Fig. 2). The overall retinal PD was derived from the average PD across the SVP, ICP, and DCP slabs.

### Statistical Analysis

All statistical analyses were conducted using SPSS Statistics 30 (IBM, Chicago, IL, USA). Demographic and general ocular outcomes were compared between emmetropes and myopes using independent samples *t*-tests, and gender distribution was compared using the χ^2^ test. Baseline SVP, ICP, and DCP PDs were examined using a linear mixed model (LMM), with the blur condition, measurement eccentricity, and perfusion layer considered as repeated within-subject factors and the refractive status considered as a between-subject factor. Significant outliers, defined as values exceeding three times the interquartile range within baseline data stratified by blur condition, measurement eccentricity, and refractive status, were excluded, resulting in the removal of 0.3% of baseline SVP and 0.4% of baseline ICP PDs from analysis. Baseline visual acuities were examined using an LMM with the blur condition used as a repeated within-subject factor and the refractive status as a between-subject factor.

Blur-induced changes in SVP, ICP and DCP PDs were calculated by subtracting the baseline values from the 60-minute measurements, followed by normalization to the baseline value to facilitate comparison of changes across different perfusion layers. These normalized changes were examined using an LMM with the blur condition, measurement eccentricity, and perfusion layer considered as repeated within-subject factors and the refractive status as a between-subject factor. Because the primary aim of the study was to compare perfusion changes between the two induced blur conditions, the changes observed in the control condition (no-blur) were included as a covariate in the LMM. Significant outliers, defined as values exceeding three times the interquartile range within PD changes stratified by blur condition, measurement eccentricity, and refractive status, were excluded, resulting in the removal of 1.6% of SVP, 0.9% of ICP, and 1.2% of DCP PD changes from analysis.

Blur-induced changes in visual acuity were also calculated by subtracting the baseline values (measured prior to blur exposure) from the 0-minute measurements (measured at the start of blur exposure) and examined using an LMM with the blur condition used as a repeated within-subject factor, the refractive status as a between-subject factor, and changes in the control condition (no-blur) considered as a covariate. A compound symmetry covariance structure was assumed for all within-participant factors included in the LMMs. Furthermore, random intercepts and slopes for individual participants were included, assuming a variance components covariance structure. Bonferroni corrected post-hoc tests were used to explore pairwise comparisons when main effects or interactions were significant.

Repeatability of PD was also examined for each retinal layer and eccentricity using the baseline and 60-minute measurements obtained during the control (no-blur) condition, following the method described by Bland and Altman.^53^ Although the 60-minute measurements were obtained after video watching with optimal refractive correction and no blur, physiological changes may still have been induced by the video viewing. Therefore, this analysis may not represent true measurement repeatability. Data are presented as mean ± standard error of the mean (SEM).

## Results

### Participant Demographics and Baseline Characteristics

Twenty-four eligible participants (67% female) with a mean age of 24 ± 1 years (range, 21–35), average SER of −0.87 ± 0.28 D (range, −5.75 to 0.50), and mean astigmatism of −0.52 ± 0.10 D (range, −1.50 to 0.00) were enrolled in the study. Participant demographic and baseline ocular characteristics, and their differences between refractive error groups, are presented in Table 1. Baseline visual acuity did not vary significantly across the blur conditions (main effect of blur, *P* = 0.083) and was −0.19 ± 0.02 logMAR in the no-blur control condition, −0.17 ± 0.02 logMAR with optical myopic defocus, and −0.17 ± 0.02 logMAR with the simulated myopic blur condition.

**Table 1.** Demographic and Baseline Ocular Characteristics of Participants

	All (*N* = 24)	Emmetropes (*N* = 12)	Myopes (*N* = 12)	*P*
Age (y), mean ± SEM	24 ± 1	26 ± 1	23 ± 1	0.118^*^
Females (%)	67	58	75	0.667^†^
Non-cycloplegic subjective refraction (D), mean ± SEM				
Spherical equivalent	−0.87 ± 0.28	0.06 ± 0.06	−1.81 ± 0.42	**<0.001** ^*^
Astigmatism	−0.52 ± 0.10	−0.33 ± 0.10	−0.71 ± 0.15	**0.046** ^*^
Optical biometry (mm), mean ± SEM				
Central corneal thickness	0.539 ± 0.007	0.532 ± 0.011	0.545 ± 0.008	0.366^*^
Anterior chamber depth	3.16 ± 0.05	3.04 ± 0.06	3.29 ± 0.06	**0.012** ^*^
Crystalline lens thickness	3.60 ± 0.05	3.66 ± 0.08	3.54 ± 0.06	0.243^*^
Axial length	23.84 ± 0.22	23.27 ± 0.16	24.41 ± 0.35	**0.009** ^*^
Baseline visual acuity (logMAR),^‡^ mean ± SEM	−0.18 ± 0.02	−0.20 ± 0.02	−0.15 ± 0.02	0.088^§^
Baseline overall retinal PD (%),^||^ mean ± SEM				
Central 3 mm	37 ± 1	37 ± 1	36 ± 1	0.438^§^
Foveal 1 mm	19 ± 1	20 ± 1	18 ± 1	**0.022** ^¶^
Parafoveal 1–2 mm	46 ± 1	46 ± 1	45 ± 1	0.779^¶^
Parafoveal 2–3 mm	45 ± 1	45 ± 1	46 ± 1	0.719^¶^
Baseline sublayer retinal PD (%), mean ± SEM				
SVP (central 3 mm)	36 ± 1	37 ± 1	36 ± 1	0.398^#^
ICP (central 3 mm)	39 ± 1	39 ± 1	38 ± 1	0.470^#^
DCP (central 3 mm)	35 ± 1	36 ± 1	35 ± 1	0.466^#^

Ocular measurements were acquired from the left eye (tested eye in this study). Statistically significant differences are indicated in bold.

* Independent samples *t*-test.

† χ^2^ test.

‡ Averaged across blur conditions given the non-significant main effect of blur (*P* = 0.083) from LMM analysis.

§ LMM, main effect of refractive status.

|| Averaged across blur conditions given the non-significant main effect of blur (*P* = 0.230) from LMM analysis.

¶ LMM, post hoc comparison from eccentricity by refractive status interaction (*P* < 0.001).

# LMM, post hoc comparison from perfusion layer by refractive status interaction (*P* = 0.889).

Similarly, baseline overall retinal PD did not differ significantly across the blur conditions (main effect of blur, *P* = 0.230) and was 36% ± 1% in the no-blur control condition, and 37% ± 1% in both the optical and simulated myopic blur conditions. Furthermore, there was no significant interaction between blur condition and perfusion layer (*P* = 0.650) or between blur condition, perfusion layer, and eccentricity (*P* = 0.976), suggesting that baseline PD across all retinal layers and eccentricities did not vary across blur conditions (Table 2).

**Table 2.** Baseline Retinal PD Across Blur Conditions

	Mean ± SEM											
	3-mm Central^*^			1-mm Fovea^†^			1- to 2-mm Inner Parafovea^†^			2- to 3-mm Outer Parafovea^†^		
Perfusion Layer	Control No Blur	Simulated Blur	Optical Blur	Control No Blur	Simulated Blur	Optical Blur	Control No Blur	Simulated Blur	Optical Blur	Control No Blur	Simulated Blur	Optical Blur
SVP (%)	36.0 ± 0.6	36.3 ± 0.6	35.8 ± 0.6	13.4 ± 0.8	14.1 ± 0.8	13.0 ± 0.8	46.1 ± 0.6	46.5 ± 0.6	45.7 ± 0.6	48.7 ± 0.6	48.2 ± 0.6	48.7 ± 0.6
*P*^‡^	—	0.595		—	0.633		—	0.217		—	0.716	
ICP (%)	38.6 ± 0.6	38.7 ± 0.6	38.7 ± 0.6	30.2 ± 0.8	29.9 ± 0.8	29.7 ± 0.8	43.8 ± 0.6	44.3 ± 0.6	44.0 ± 0.6	41.8 ± 0.6	41.8 ± 0.6	42.2 ± 0.6
*P*^‡^	—	>0.99		—	>0.99		—	>0.99		—	0.964	
DCP (%)	34.8 ± 0.6	35.5 ± 0.6	35.1 ± 0.6	13.1 ± 0.8	13.8 ± 0.8	12.8 ± 0.8	46.1 ± 0.6	47.0 ± 0.6	46.6 ± 0.6	45.3 ± 0.6	45.6 ± 0.6	46.0 ± 0.6
*P*^‡^	—	0.938		—	0.729		—	0.876		—	>0.99	

Ocular measurements are acquired from the left eye (tested eye in this study).

* LMM, interaction between blur condition and perfusion layer (*P* = 0.650).

† LMM, interaction among blur condition, perfusion layer, and eccentricity (*P* = 0.976).

‡ LMM, post hoc comparison between simulated and optical myopic blur conditions.

### Repeatability of PD

Retinal PD did not change significantly across any perfusion layer during the no-blur control condition (all *P* > 0.05). The coefficient of repeatability for PD measurements obtained during the control session ranged from 2.7% to 8.3% for the SVP, 2.2% to 4.0% for the ICP, and 2.2% to 3.4% for the DCP (Table 3).

**Table 3.** Repeatability of PD Measurements During the No-Blur Control Condition

	1-mm Fovea			1- to 2-mm Inner Parafovea			2- to 3-mm Outer Parafovea		
Perfusion Layer (%)	Mean Difference ± SD	CR	95% CI	Mean Difference ± SD	CR	95% CI	Mean Difference ± SD	CR	95% CI
SVP	0.1 ± 1.4	2.7	2.4–3.0	0.5 ± 2.6	5.1	4.5–5.6	0.5 ± 4.3	8.3	7.5–9.2
ICP	−0.1 ± 2.1	4.0	3.6–4.4	0.4 ± 1.1	2.2	2.0–2.4	0.2 ± 1.2	3.8	3.4–4.2
DCP	−0.2 ± 1.8	3.4	3.1–3.8	−0.1 ± 1.1	2.2	1.9–2.4	0.4 ± 1.4	3.3	2.9–3.6

CR, coefficient of repeatability.

### Blur-Induced Changes in Visual Acuity

There was no significant main effect of blur type on visual acuity changes (*P* = 0.181), with visual acuity reduced by a similar amount during the digitally simulated (0.63 ± 0.02 logMAR) and optical myopic blur (0.60 ± 0.02 logMAR) conditions.

### Blur-Induced Changes in PD Averaged Across All Retinal Layers

There was no significant main effect of blur on retinal PD changes when averaged across all perfusion layers and eccentricities (*P* = 0.221). However, a significant blur by eccentricity interaction was observed (*P* < 0.001), suggesting regional variations in the blur-induced PD changes. Retinal PD increased in response to optical compared to simulated myopic blur in the foveal region (2.9% ± 1.0%; *P* = 0.003) but decreased significantly with optical compared to simulated myopic blur in the outermost 2- to 3-mm parafoveal region (−2.3% ± 0.5%; *P* < 0.001).

**Figure 3.**
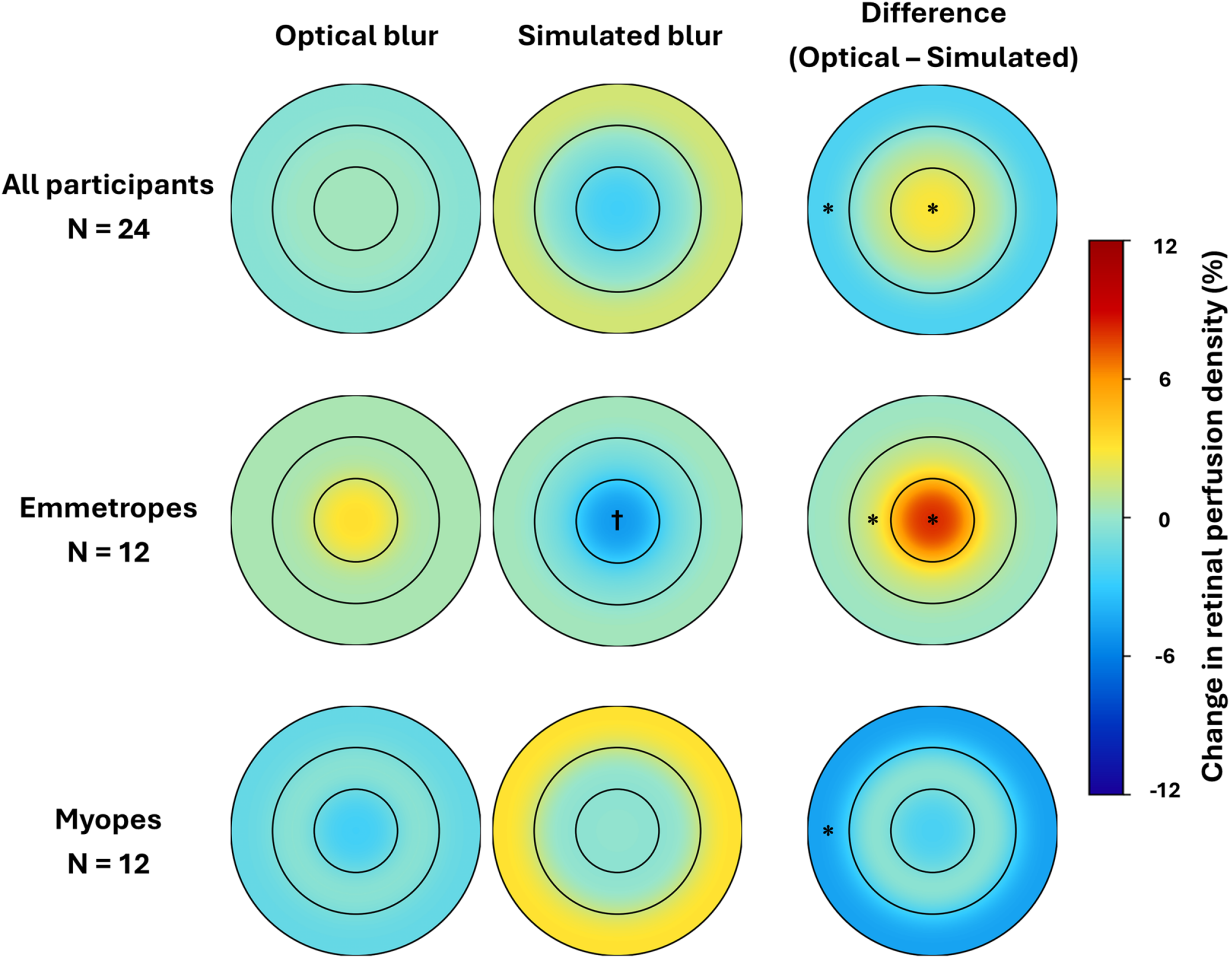
Changes in retinal PD averaged across all layers following short-term exposure to optical myopic blur (*left column*) and digitally simulated myopic blur (*middle column*), along with the difference in PD changes between the two blur conditions (*right column*). Results are derived from LMM analysis and are illustrated for all participants (*top row*), emmetropes (*middle row*), and myopes (*bottom row*). Blur-induced changes in PD were adjusted for each participant's change during the no-blur control condition. Eccentricity zones include the central 1-mm fovea and the surrounding inner (1- to 2-mm annulus) and outer (2- to 3-mm annulus) parafovea. ^∗^Significant difference between optical and simulated myopic blur (*P* < 0.05). ^†^Significant difference relative to the control condition at the corresponding eccentricity (*P* < 0.05), based on the blur by eccentricity interaction (*P* < 0.001, *top row*) and the blur by eccentricity by refractive status interaction (*P* < 0.001, *middle* and *bottom rows*).

A significant interaction among blur, eccentricity, and refractive status was also observed (*P* < 0.001). Short-term exposure to optical blur significantly increased retinal PD in emmetropes compared with exposure to simulated blur, at both the fovea (8.2% ± 1.4%; *P* < 0.001) and 1- to 2-mm parafoveal (1.5 ± 0.7%; *P* = 0.03) eccentricities. Furthermore, short-term exposure to simulated blur significantly decreased PD in emmetropes compared to the no-blur control condition at the fovea (−5.0% ± 1.7%; 95% confidence interval [CI], −9.9 to −0.10). In contrast, exposure to optical blur decreased retinal PD in myopes compared with simulated blur, primarily in the outermost 2- to 3-mm parafoveal region (−4.6% ± 0.8%; *P* < 0.001) (Fig. 3).

### Blur-Induced Changes in PD Across Each Retinal Layer

**Figure 4.**
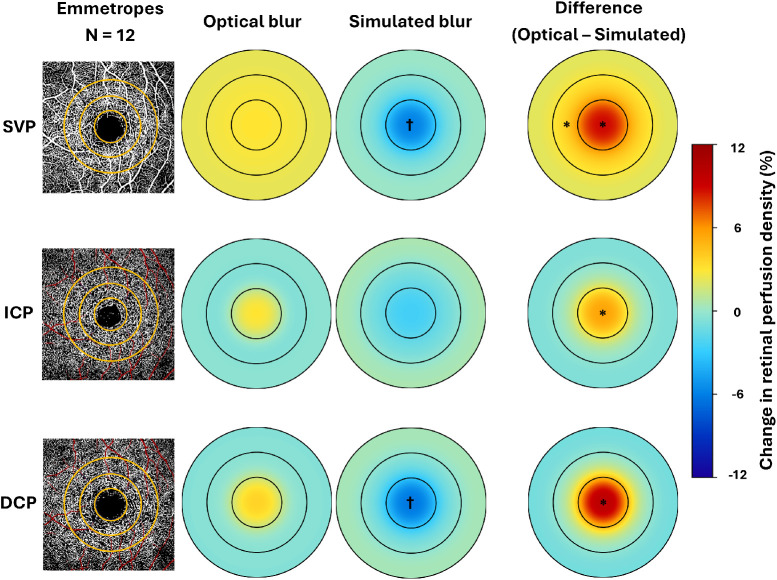
Changes in retinal PD in emmetropes following short-term exposure to optical myopic blur (*left column*) and simulated myopic blur (*middle column*), along with the difference in PD changes between the two blur conditions (*right column*). Results are derived from LMM analysis and are illustrated across the SVP (*top row*), ICP (*middle row*), and DCP (*bottom row*). Blur-induced changes in PD were adjusted for each participant's change during the no-blur control condition. Eccentricity zones include the central 1-mm fovea and the surrounding inner (1- to 2-mm annulus) and outer (2- to 3-mm annulus) parafovea. ^∗^Significant difference between optical and simulated myopic blur (*P* < 0.05). ^†^Significant difference relative to the control condition at the corresponding eccentricity (*P* < 0.05), based on interaction among blur, eccentricity, perfusion layer, and refractive status (*P* = 0.04).

A significant interaction among blur, eccentricity, perfusion layer, and refractive status was observed (*P* = 0.04), suggesting that blur-induced regional changes in retinal PD varied across both vascular layers and refractive groups (Table 4). In emmetropes, short-term exposure to optical myopic blur significantly increased the foveal PD compared with simulated myopic blur across all perfusion layers (all *P* < 0.05). Furthermore, exposure to simulated myopic blur significantly reduced the foveal PD in emmetropes compared to the no-blur control condition in the SVP (−6.0% ± 2.0%; 95% CI, −10.9 to −1.10) and DCP layers (−6.4% ± 2.2%; 95% CI, −11.3 to −1.50) (Fig. 4).

**Table 4.** Blur-Induced Changes in Retinal PD in Emmetropic and Myopic Eyes

	1-mm Fovea			1- to 2-mm Inner Parafovea			2- to 3-mm Outer Parafovea		
Perfusion Layer	Simulated Blur, Mean ± SEM	Optical Blur, Mean ± SEM	*P* ^*^	Simulated Blur, Mean ± SEM	Optical Blur, Mean ± SEM	*P* ^*^	Simulated Blur, Mean ± SEM	Optical Blur, Mean ± SEM	*P* ^*^
SVP (%)									
Emmetropes (*n* = 12)	−6.0 ± 2.2	3.0 ± 2.3	**<0.001**	−0.9 ± 1.7	2.7 ± 1.7	**0.002**	0.1 ± 1.7	2.3 ± 1.8	0.086
Myopes (*n* = 12)	−3.2 ± 2.3	−0.8 ± 2.3	0.329	0.8 ± 1.8	0.3 ± 1.8	0.705	4.9 ± 1.7	−2.6 ± 1.7	**<0.001**
*P*^†^	0.399	0.261	—	0.526	0.385	—	0.128	0.121	—
ICP (%)									
Emmetropes (*n* = 12)	−2.6 ± 2.2	3.1 ± 2.4	**0.022**	−1.1 ± 1.7	−0.8 ± 1.7	0.799	0.5 ± 1.7	−0.2 ± 1.7	0.537
Myopes (*n* = 12)	1.8 ± 2.4	−3.0 ± 2.3	0.06	−0.7 ± 1.7	−1.0 ± 1.8	0.813	2.9 ± 1.7	−1.5 ± 1.7	**<0.001**
*P*^†^	0.209	0.09	—	0.886	0.929	—	0.382	0.645	—
DCP (%)									
Emmetropes (*n* = 12)	−6.4 ± 2.2	3.8 ± 2.3	**<0.001**	−0.9 ± 1.7	−0.3 ± 1.7	0.598	0.4 ± 1.7	−0.6 ± 1.7	0.404
Myopes (*n* = 12)	0.75 ± 2.3	−4.0 ± 2.3	0.053	−1.1 ± 1.7	0.2 ± 1.7	0.297	1.6 ± 1.8	−0.4 ± 1.7	0.123
*P*^†^	**0.046**	**0.035**	—	0.96	0.831	—	0.659	0.95	—

Blur-induced changes in PD were adjusted for each participant's change during the no-blur control condition.

* LMM, Bonferroni-corrected post hoc comparison between blur conditions.

† LMM, Bonferroni-corrected post hoc comparison between emmetropes and myopes.

**Figure 5.**
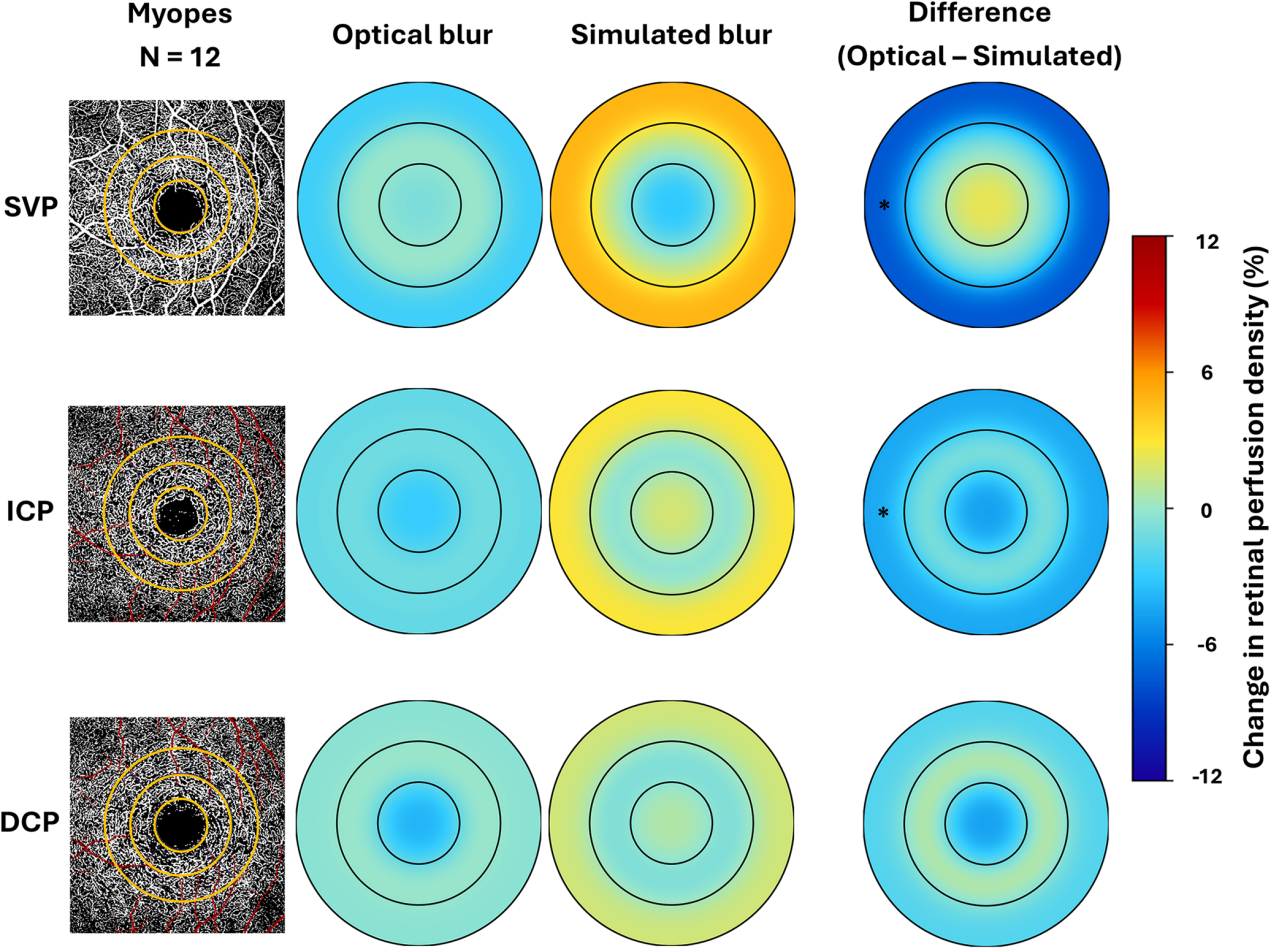
Changes in retinal PD in myopes following short-term exposure to optical myopic blur (*left column*) and simulated myopic blur (*middle column*), along with the difference in PD changes between the two blur conditions (*right column*). Results are derived from LMM analysis and illustrated across the SVP (*top row*), ICP (*middle row*), and DCP (*bottom row*). Blur-induced changes in PD were adjusted for each participant's change during the no-blur control condition. Eccentricity zones include the central 1-mm fovea and the surrounding inner (1- to 2-mm annulus), and outer (2- to 3-mm annulus) parafovea. ^∗^Significant difference between optical and simulated myopic blur (*P* < 0.05). ^†^Significant difference relative to the control condition at the corresponding eccentricity (*P* < 0.05), based on interaction among blur, eccentricity, perfusion layer, and refractive status (*P* = 0.04).

In contrast, myopes exhibited a significant reduction in PD in response to optical compared to simulated myopic blur in the 2- to 3-mm parafoveal region across the SVP and ICP (both *P* < 0.05). This finding appeared to be driven by a paradoxical increase in PD of the 2- to 3-mm parafoveal region in response to simulated blur in myopes (Fig. 5).

**Figure 6.**
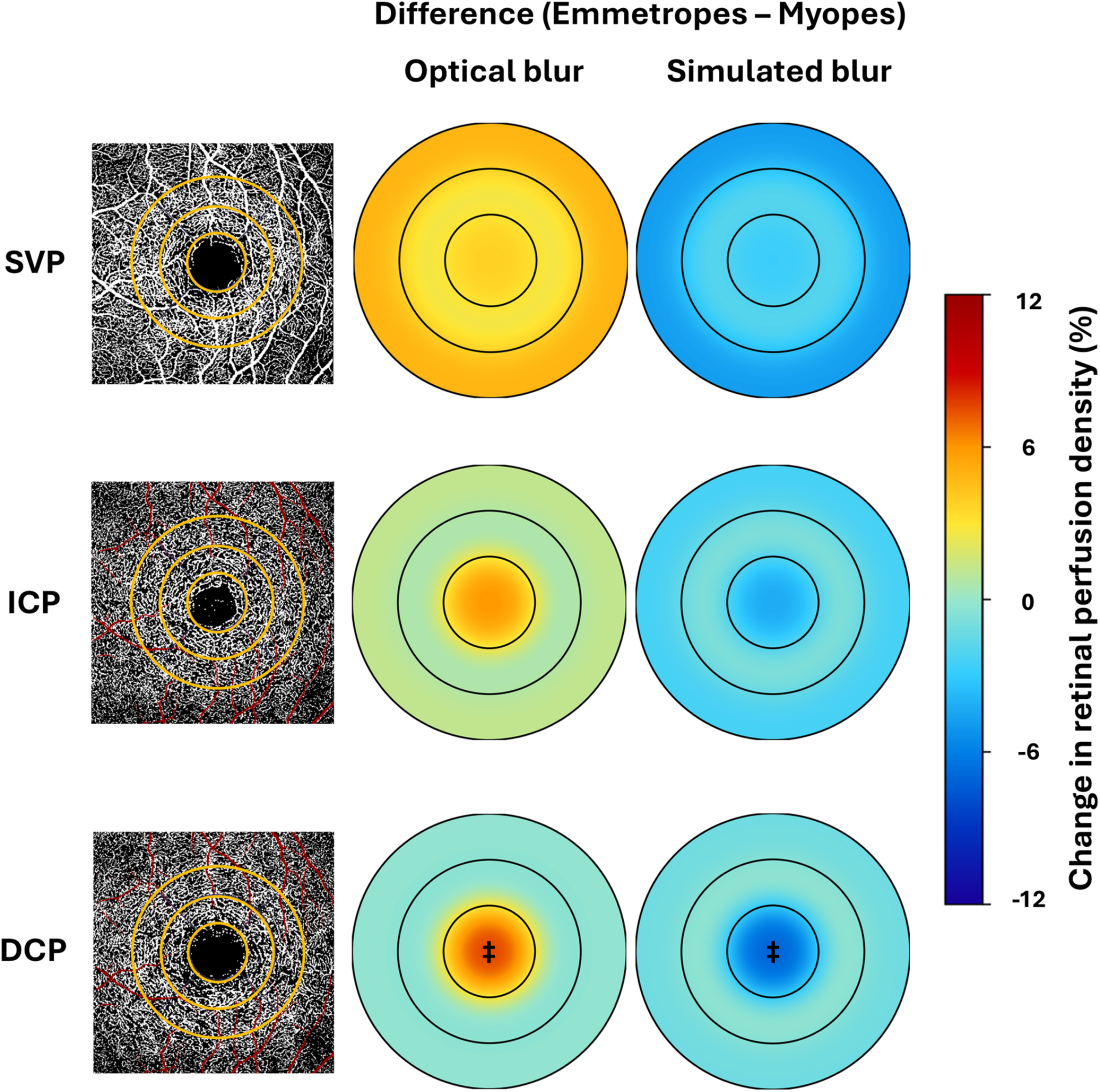
The difference in retinal PD changes between emmetropes and myopes following short-term exposure to optical myopic blur (*left column*) and simulated myopic blur (*right column*). Results are derived from LMM analysis and illustrated across the SVP (*top row*), ICP (*middle row*), and DCP (*bottom row*). Blur-induced changes in PD were adjusted for each participant's change during the no-blur control condition. Eccentricity zones include the central 1-mm fovea and the surrounding inner (1- to 2-mm annulus) and outer (2- to 3-mm annulus) parafovea. ^‡^Significant difference in blur-induced PD change between emmetropes and myopes at the corresponding eccentricity (*P* < 0.05), based on interaction among blur, eccentricity, perfusion layer, and refractive status (*P* = 0.04).

The largest refractive group–dependent differences in retinal perfusion responses to blur were observed in the DCP layer at the fovea. Short-term exposure to optical myopic blur increased the foveal DCP PD in emmetropes (3.8% ± 2.3%) but decreased it in myopes (−4.0% ± 2.3%; *P* = 0.035). Conversely, short-term exposure to digitally simulated myopic blur reduced the foveal DCP PD in emmetropes (−6.4% ± 2.1%) but increased it in myopes (0.75% ± 2.3%; *P* = 0.046) (Fig. 6).

## Discussion

This study provides the first, to our knowledge, comprehensive evidence on retinal perfusion changes induced by short-term exposure to different types of blur in the human eye. When image blur was optically induced by +2 D lenses to create optical myopic defocus on the retina, a relative increase in foveal PD and a relative reduction in outer parafoveal PD were observed, when compared with digitally simulated myopic blur of comparable magnitude. Furthermore, these blur-driven retinal perfusion changes were associated with refractive error status. Emmetropic eyes demonstrated an overall increase in the PD in response to optical myopic defocus and a decrease in response to simulated myopic blur, with these changes being more pronounced in the fovea. In contrast, myopic eyes did not exhibit a corresponding increase in foveal PD in response to optical myopic defocus but instead demonstrated increased outer parafoveal perfusion with simulated myopic blur.

Further analysis revealed that these refractive-dependent variations in PD in response to different blur types originated primarily from the DCP located near the synaptic region between photoreceptors and bipolar and horizontal cells, where emmetropes exhibited a significantly greater increase in PD with optical myopic defocus and a greater reduction with simulated myopic blur, compared with myopes. Collectively, these findings provide novel insights into short-term retinal perfusion changes induced by blur that may reflect differences in the way that emmetropic and myopic eyes process blur and may ultimately contribute to long-term ocular growth and the development of myopia.

### Retinal Perfusion Response to Optical Myopic Defocus

Swiatczak et al.^33^ were the first group to examine ocular blood flow responses to optical myopic defocus in young human subjects using LSFG. Following a 30-minute exposure to myopic defocus, no blood flow changes were observed at the optic nerve head, where the LSFG signal is primarily determined by retinal circulation. In contrast, a significant increase in blood flow was observed in the macular region, where the LSFG signal is primarily contributed by choroidal circulation. These findings led them to conclude that optical myopic defocus selectively increases choroidal, but not retinal, blood flow.^22^^,^^33^

In the current study, OCTA imaging was used, which does not measure blood flow but instead characterizes retinal perfusion with high depth-resolution across superficial vessels, as well as intermediate and deep capillaries in the retina, without signal contamination from choroidal perfusion. In contrast to the work of Swiatczak et al.,^33^ the present findings revealed that retinal perfusion is responsive to optical myopic defocus, with this response also being associated with the refractive status of the eye.

In emmetropic eyes, increased retinal perfusion was observed in the foveal region of the DCP (3.8%) and ICP (3.1%), as well as in the foveal (3.0%) and inner parafoveal (2.7%) regions of the superficial (inner) vascular plexus in response to imposed optical myopic defocus. These changes were also clinically meaningful, particularly in the foveal region, as they exceeded repeatability limits. There is currently limited evidence regarding the magnitude of retinal PD changes induced by defocus or other visual environmental factors relevant to myopia. However, the magnitude of the changes observed in the present study appears comparable to, or greater than, that reported in previous OCTA studies. For example, in pre-myopic children, DCP PD across the central 6 mm of the macula increased by 3.2% following 6 months of red laser light therapy.^41^ A short-term study in healthy older adults found increases in PD of ∼4% in the DCP and ∼6% in the SVP following brief (∼1 minute) exposure to flickering light.^54^ Other studies have reported relatively modest retinal perfusion changes, including small-magnitude, non-significant increases of ∼0.1% to 2% in the deep and superficial capillary plexuses after 1 hour of reading at 33 cm in children^47^ and young adults.^55^ Another study found a non-significant ∼0.25% increase in DCP PD, coupled with a significant local parafoveal reduction of ∼2.0% in SVP PD following 1 hour of 0.1% atropine administration in young adults.^56^ Taken together, these findings suggest that the retinal perfusion changes observed in the present study are within a physiologically plausible range and of a magnitude similar to, or greater than, previously reported retinal perfusion responses associated with myopia-related visual interventions.

These OCTA findings are consistent with a previous study on primates demonstrating that responses of ON-bipolar and GABAergic amacrine cells in the retina are enhanced by short-term exposures to myopically defocused images compared to hyperopically defocused or diffusely blurred images.^57^ Electrophysiological studies in humans have also found that reduced inner retinal neural responses in the central 1.6° region were a risk factor for greater eye growth and myopia progression in myopic children wearing single-vision spectacles, whereas this risk factor was not evident in myopic children treated with orthokeratology lenses.^58^ Enhanced inner retinal neural responses also occurred with short-term exposure to optical defocus, with more pronounced changes observed in the 6° to 12° macular region in response to full-field myopic defocus,^59^ but more evenly distributed changes were observed across the central 18° macular region in response to dual-focus concentric myopia control contact lenses.^60^ The present OCTA data did not show a corresponding dominance of increased inner retinal PD in the outer parafovea in response to full-field optical myopic defocus, possibly because imaging was limited to the central 10° region. Future OCTA studies covering a wider retinal area are therefore warranted to determine whether these myopic defocus-driven changes in retinal perfusion extend beyond 10°.

As demonstrated in the present study, retinal perfusion in the inner retinal layers increases alongside the elevated electrophysiological activity reported by Ho et al.^59^ in response to short-term exposure to optical myopic defocus. These findings lend support to neurovascular coupling in the retina during the processing of optical myopic defocus. They further support a study in animals demonstrating upregulation of cytochrome *c* oxidase in the retina in response to optical myopic defocus,^61^ suggesting that processing of myopic defocus is metabolically demanding for the retina.

Furthermore, the current results have provided novel insights, suggesting that the optical myopic defocus-induced increase in retinal perfusion is impaired in myopic eyes, particularly within the central 1-mm (∼3°) region of the DCP. In this region, a −4% reduction in PD was observed in myopes in response to optical myopic defocus, as opposed to a 3.8% increase in emmetropes, representing both statistically and clinically significant differences. The DCP corresponds to retinal layers where photoreceptors synapse with bipolar and horizontal cells in the inner nuclear layer.^51^ Photoreceptors receive their metabolic support primarily from the choriocapillaris in the choroid, whereas bipolar and horizontal cells are mainly supplied by the DCP in the retina.^62^ Therefore, myopic eyes may exhibit impaired processing of myopic defocus within the inner retina, as demonstrated by the absence of increased perfusion in the DCP that was evident in emmetropic eyes.

This interpretation aligns with recent psychophysical evidence showing that myopes have impaired blur adaptation to myopic defocus, likely associated with altered ON-pathway signaling originating from the inner nuclear layer of the retina.^49^ The lack of foveal perfusion changes in myopes in response to optical myopic defocus is also consistent with several studies reporting attenuated axial length responses to imposed optical myopic defocus in myopic eyes.^11^^,^^12^^,^^63^ Future studies examining changes in choriocapillaris perfusion in response to optical myopic defocus are warranted to provide further insights into photoreceptor processing of myopic defocus in myopic eyes. Future longitudinal studies could also examine short-term optical myopic defocus-induced changes in retinal perfusion in children to determine whether impaired retinal perfusion responses would precede the development of myopia or arise after myopia onset.

### Retinal Perfusion Response to Digitally Simulated Myopic Blur

Short-term exposure to simulated myopic blur of comparable magnitude to the optical myopic defocus condition resulted in a distinct retinal perfusion response, with a statistically and clinically significant decrease in retinal PD observed in emmetropic eyes, primarily within the central foveal region of the DCP (−6.4%) and SVP (−6.0%). This finding is consistent with a recent electrophysiological study showing reduced amplitudes of retinal responses to simulated blur imposed across a 12° visual field (equivalent to ∼0.2 D of blur for a 5-mm pupil).^64^ Therefore, the simulated blur-induced reductions in retinal PD in emmetropic eyes are likely mediated by neurovascular coupling.^31^ Interestingly, the electrophysiological amplitude reductions with simulated blur have been predominantly observed in responses originating at the level of ganglion cells and, to a lesser extent, bipolar cells.^64^ In contrast, the present study demonstrated PD reductions in both the SVP and DCP, corresponding to the ganglion cell and bipolar cell layers. The greater magnitude of simulated myopic blur used in the current study (2 D vs. ∼0.2 D) may have contributed to the stronger PD reductions observed across both the inner and outer retina.

The retinal perfusion response of myopic eyes to simulated myopic blur differed from that of emmetropic eyes and was characterized by a small magnitude (2.9% and 4.9% in the ICP and SVP, respectively) but statistically significant relative increase in PD in the outer parafoveal region (∼7°–10°) compared with the response to optical myopic defocus, rather than the reduction in perfusion observed in emmetropes. This finding may reflect differences in blur sensitivity between emmetropes and myopes, consistent with psychophysical studies reporting reduced sensitivity to peripheral simulated blur in myopes compared with emmetropes.^65^ A reduced sensitivity of myopic eyes to peripheral simulated blur may explain the absence of perfusion reduction in response to simulated myopic blur observed in the present study, assuming that neurovascular coupling is not impaired in myopic eyes. However, a limitation of this study was that the visual field coverage of the simulated myopic blur stimulus (9° horizontally and 5° vertically) was smaller than the OCTA scan area of the outer parafoveal 2- to 3-mm annulus ring (outer edge ∼10°), effectively representing about 40% coverage of this annulus. Consequently, the outer annulus in the OCTA images may not have reliably represented PD changes in response to the simulated blur and may have also been influenced by responses to the clear peripheral stimuli surrounding the display monitor.

Short-term exposure to simulated myopic blur increases axial length in both emmetropic and myopic eyes, thus implicating exposure to a low-pass-filtered visual environment as a contributing factor to myopia progression in both refractive groups, with changes in retinal perfusion playing an important role. This finding is consistent with animal studies using low-pass-filtered movies to induce myopia,^66^ and with modeling of the spatial frequency spectrum from images of different visual environments, which suggest that reduced energy at higher spatial frequencies may contribute to the high prevalence of myopia in industrialized countries.^67^

### Potential Mechanisms to Distinguish Optical From Simulated Myopic Blur

In this study, distinct retinal perfusion responses were observed for different blur types, particularly in emmetropic eyes, despite both optical and simulated myopic blur selectively reducing contrast at high spatial frequencies and reducing visual acuity to a similar level. Previous studies have shown that retinal electrophysiological responses decrease with reductions in mean luminance contrast and with low-pass filtering of spatial frequencies in images,^68^^,^^69^ which may explain the reduced PD with simulated myopic blur observed in the current study but does not explain the opposite response (increased PD) observed to optical myopic defocus (which similarly affects contrast and spatial frequency content). The differential responses observed between optical and simulated myopic blur in the present study may therefore be related to the presence of additional light vergence cues during exposure to optical myopic defocus induced by positive lenses, which could have counteracted the effects of selective contrast reductions at high spatial frequencies.

It is possible that photoreceptor light capture may have been enhanced through phototropic mechanisms during exposure to optical myopic defocus, with re-alignment of photoreceptors to the direction of maximum light intensity.^70^ This light-driven photoreceptor adjustment could increase neuronal activity in the outer retina, thereby elevating metabolic demand and oxygen consumption. The resulting increase in the outer retinal energy requirement would likely involve changes in choriocapillaris perfusion, which primarily supplies photoreceptors, but requires future studies to confirm. Enhanced outer retinal energy requirement may also stimulate greater neuronal activity in downstream inner retinal circuits, reflected by the observed increase in perfusion of the DCP. In contrast, changes in photoreceptor light capture are unlikely during exposure to simulated myopic blur, given the absence of light vergence cues. This may explain the reduced perfusion observed in the DCP in response to simulated blur in emmetropic eyes, most likely explained by selectively attenuated contrast at high spatial frequencies.

Both light vergence and binocular vergence are modified by optical defocus. Optical myopic defocus increases light vergence and, when induced binocularly, results in dis-accommodation and divergence. In contrast, optical hyperopic defocus decreases light vergence and, when induced binocularly, induces accommodation and convergence. However, in the current study, binocular vergence cues were not expected to differ substantially between the optical and simulated myopic blur conditions. This is because accommodation primarily responds to light vergence changes rather than to digitally simulated blur.^71^ Furthermore, any binocular divergence resulting from dis-accommodation under optical myopic defocus would likely be minimal in the current study, given that optimal refractive correction was provided for a far viewing distance. Therefore, it is unlikely that binocular vergence cues substantially contributed to the differential retinal PD changes observed in response to optical and simulated myopic blur in the current study.

## Conclusions

In this study, increased foveal retinal perfusion following short-term exposure to optical myopic defocus but decreased perfusion following short-term exposure to digitally simulated myopic blur were identified as characteristic responses in healthy young adult emmetropic eyes, whereas these responses appeared to be altered in myopic eyes. These distinct retinal perfusion responses to different blur types were observed despite similar reductions in visual acuity under the imposed optical and simulated myopic blur conditions, suggesting that mechanisms other than image blur magnitude, or contrast and spatial frequency modulation, may be involved. The primary difference between the blur conditions used in this study are the light vergence cues provided by optical myopic defocus that are absent in digitally simulated myopic blur. Given the potential role of ocular hypoxia in myopia development and the contribution of the choroid to both hypoxia- and vision-dependent mechanisms of eye growth,^72^ future studies are required to examine choroidal perfusion changes with growth inhibitory stimuli, such as optical myopic defocus, and growth stimulatory stimuli, such as digitally simulated blur and optical hyperopic defocus, to further elucidate the hemodynamic mechanisms underlying myopia.

Furthermore, the present study was cross-sectional and conducted in young adults with established myopia. Therefore, it does not provide evidence for a causal link between altered blur-induced retinal perfusion responses and the development or progression of myopia, which primarily occurs during childhood. The observed differences may instead reflect changes associated with myopia development, rather than mechanisms involved in the onset or progression of myopia. Consequently, future longitudinal studies in pediatric populations are warranted to determine whether these retinal perfusion responses to blur inform the early development or progression of myopia.
